# IFITM3‐specific antibody reveals IFN preferences and slow IFN induction of the antiviral factor IFITM3 in humans

**DOI:** 10.1002/eji.202048706

**Published:** 2020-12-09

**Authors:** Dannielle Wellington, Zixi Yin, Adi Abdel‐Haq, Liwei Zhang, Jessica Forbester, Kerry Kite, Ushani Rajapaksa, Henry Laurenson‐Schafer, Shokouh Makvandi‐Nejad, Boquan Jin, Emma Bowes, Krishnageetha Manoharan, David Maldonado‐Perez, Clare Verrill, Ian R. Humphreys, Tao Dong

**Affiliations:** ^1^ MRC Human Immunology Unit, MRC Weatherall Institute of Molecular Medicine, Radcliffe Department of Medicine Oxford University Oxford UK; ^2^ Nuffield Department of Medicine Chinese Academy of Medical Sciences (CAMS) Oxford Institute Oxford University Oxford UK; ^3^ Charles Tanford‐Proteinzentrum, Martin‐Luther‐Universität Halle‐Wittenberg Institut für Molekulare Medizin Kurt‐Mothes‐Straße 3a Halle Germany; ^4^ Division of Infection and Immunity/Systems Immunity University Research Institute Cardiff University Cardiff UK; ^5^ Fourth Military Medical University Xian China; ^6^ Oxford Radcliffe Biobank, Nuffield Department of Surgical Sciences Oxford University Oxford UK

**Keywords:** IFITM3, IFN, myeloid cells, innate immunity, viral restriction

## Abstract

Using a specific antibody, we found that expression of the viral restriction factor IFITM3 differs across cell types within the immune compartment with higher expression in myeloid rather than lymphoid cells. IFITM3 expression was increased following IFN stimulation, mostly type I, in immune cells, with the exception of T cells.

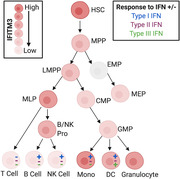

Interferon (IFN) induced trans‐membrane protein 3 (IFITM3) has been shown to restrict replication of around seventeen, mostly enveloped, RNA viruses including influenza A virus (IAV), HIV‐1, Ebola, SARS coronavirus, and Dengue virus [1, 2]. The tropism of these viruses is highly varied; HIV‐1 predominantly infects CD4^+^ T cells, IAV predominantly epithelial cells of the respiratory system and Dengue virus can infect skin and Langerhans cells.

The mechanisms of IFITM3‐mediated viral restriction have yet to be determined. Investigations into IFITM3 restriction have been hindered by a lack of antibodies specific to IFITM3 as most commercially available antibodies cross‐react to the closely related homolog IFITM2.

We custom‐generated an IFITM3‐specific antibody with minimal cross‐reactivity to IFITM2 [3]. Using this antibody and CRISPR‐generated IFITM3^−/−^ human cell lines, we found differences in basal expression of IFITM3 across several cell lines, with IFITM3 expression decreasing following differentiation of induced pluripotent stem cells (iPSC) into macrophages (iPSC‐Mac), in line with previous reports [4] (Supporting Information Fig. S1).

Investigation into the basal expression pattern of IFITM3 in primary human samples revealed differences across immune cell subsets. There was a consistent pattern of higher expression in myeloid compared to lymphoid cells in adult blood samples (*p* = 0.0202), lung para‐tumor tissue (*p* = 0.0007), and cord blood samples (*p* = 0.0175; Fig. 1A).

**Figure 1:**
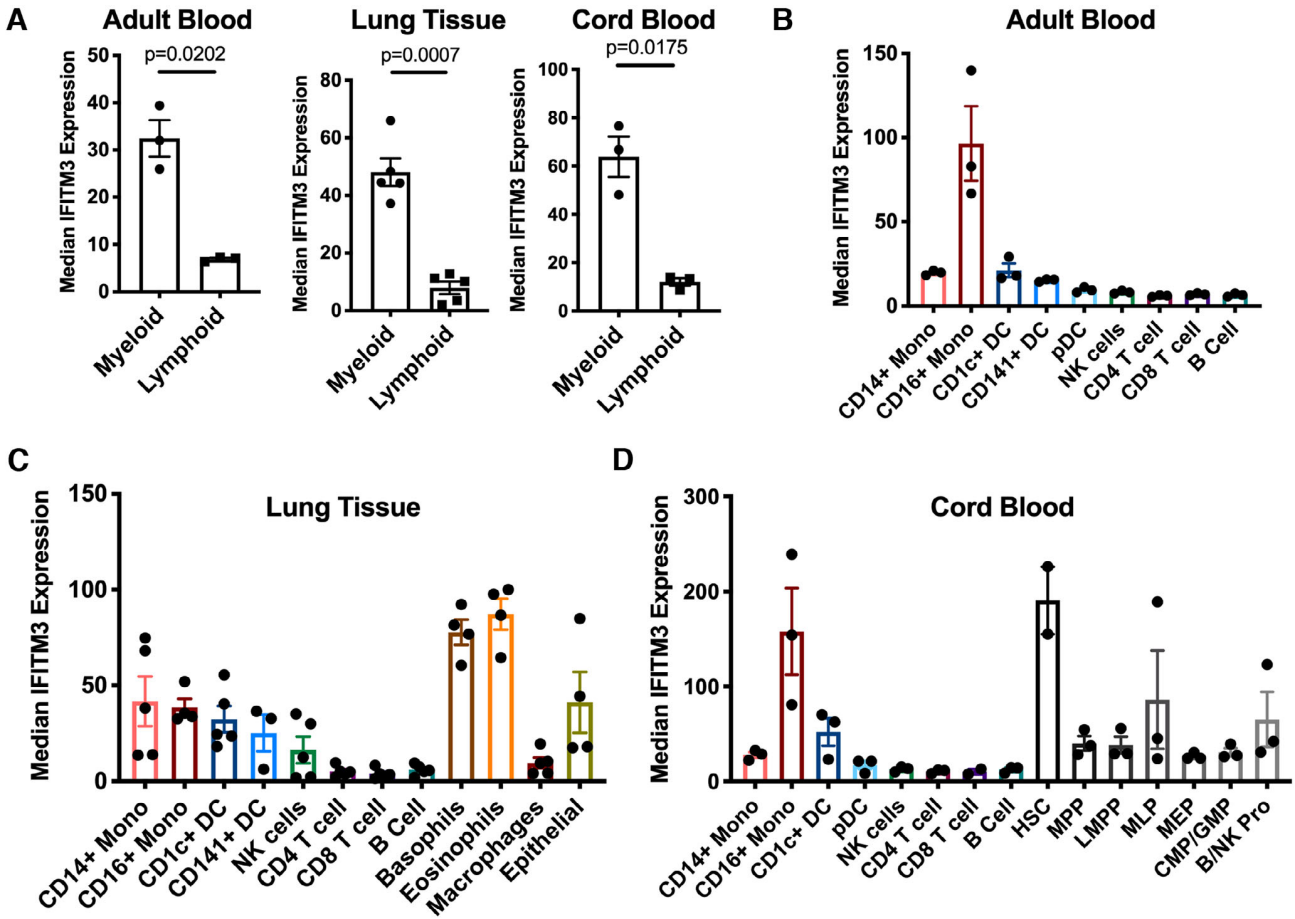
**Basal IFITM3 expression varies in primary human cells**. IFITM3 expression was measured in primary human samples using CyTOF. (A) Results from adult blood, lung para‐tumor tissue and cord blood samples. Expression in myeloid and lymphoid cells was compared by paired *t*‐test. (B) Basal IFITM3 expression in human adult blood samples shown by individual cell types. (C) Basal IFITM3 expression in human lung para‐tumor tissue shown by individual cell types. (D) Basal IFITM3 expression in human cord blood samples shown by individual cell types. HSC, hematopoietic stem cells; MPP, multipotent progenitor; LMPP, lymphoid‐primed multipotent progenitors; MLP, multi‐lymphoid progenitor; MEP, megakaryocyte erythroid progenitor; CMP, common myeloid progenitor; GMP, granulocyte‐monocyte progenitors; B/NK Pro, B cell + NK cell progenitor. Data are expressed ±SEM with mean center values. Adult blood donors *n* = 3, one experiment, lung tissue samples *n* = 5, four independent experiments, Cord blood samples *n* = 3, three independent experiments.

IFITM3 was highest in CD16^+^ monocytes from adult blood (Fig. 1B), while expression in the lung was highest in granulocyte populations (Fig. 1C). The lung para‐tumor samples allowed us to investigate the expression of IFITM3 in lung epithelial cells, most likely type 1 and 2 pneumocytes, however we were unable to identify ciliated epithelial cells as these are present higher up the respiratory tract, preventing direct comparison to previous mouse models [5].

In cord blood samples, IFITM3 expression was highest in hematopoietic stem cells (HSC) in accordance with previous suggestions [4]. However, the high expression in CD16^+^ monocytes in these samples suggests that it is not as simple as increased differentiation leading to reduced expression compared to HSC.

The pattern of expression across immune cell subsets was not shared by other IFN‐stimulated genes (ISG) investigated here, STAT1 or BST2, that are also involved in antiviral responses [6, 7] (Supporting Information Fig. S2). However, there was higher expression in myeloid compared to lymphoid cells for both proteins.

As an ISG, IFITM3 has previously been shown to be induced by both type I and type II IFNs in a murine setting [5]. We stimulated HEK293 and A549 human cell lines with 0–10,000 U/mL IFN for 24 h and measured IFITM3 expression by western blot (Supporting Information Fig. 3). We saw strong responses to type I IFNs but a much weaker response to type II and III IFNs, with some differences across the two cell lines.

In primary immune cells, there was some induction with type I IFN but minimal response to other types of IFN and these results were not significant (Fig. 2A). When the immune cells were separated into myeloid and lymphoid cells there was again a strong induction with type I IFN in myeloid cells (*p* = 0.0449) by IFN‐α, but negligible increases were seen in the lymphoid compartment (Fig. 2B). After 48 h IFN stimulation there was no measurable increase in STAT1 or BST2 suggesting that these proteins had already peaked and returned to baseline levels by 48 h (data not shown).

**Figure 2:**
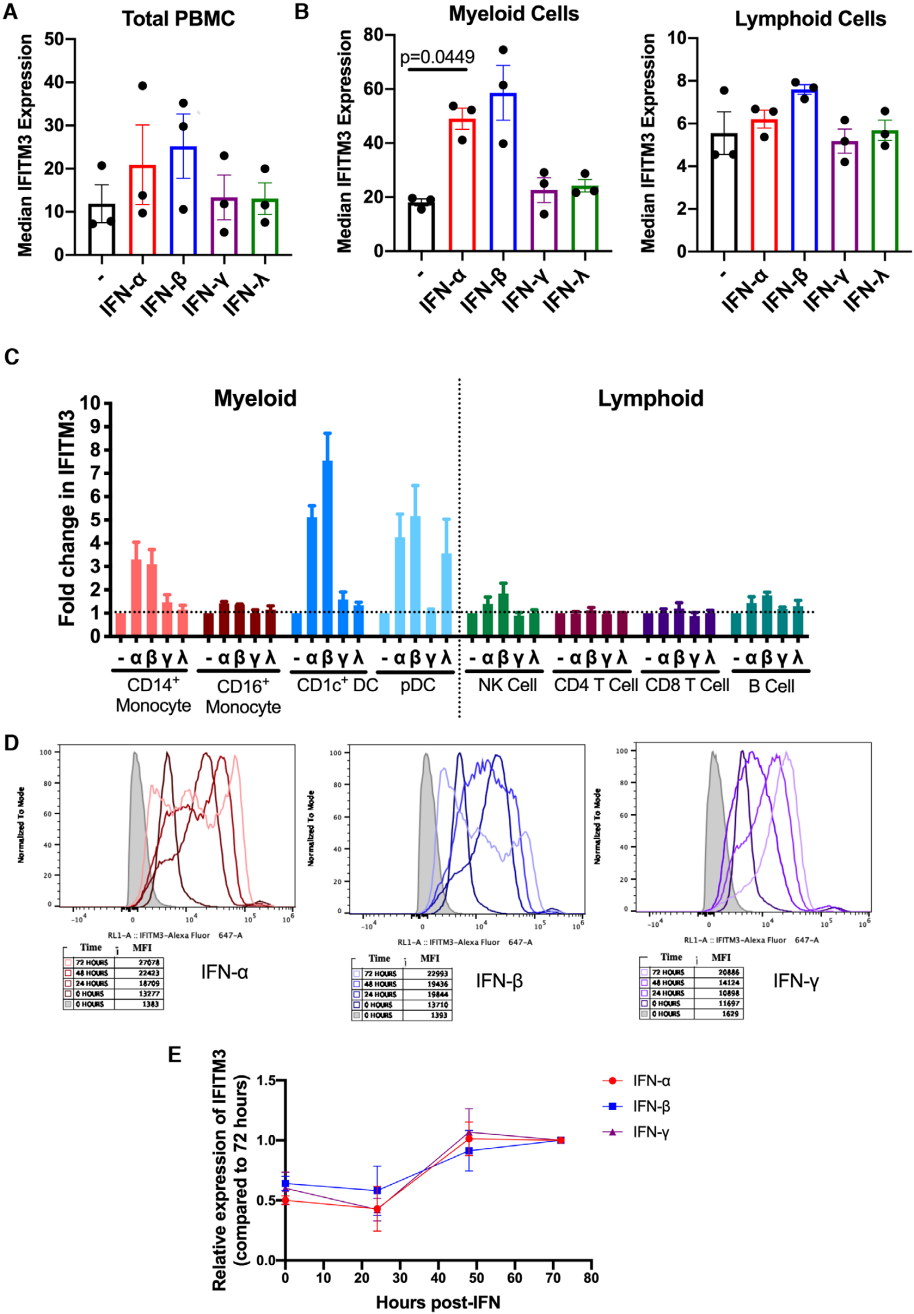
**IFN induction of IFITM3 in primary human cells**. (A) Expression of IFITM3 was measured by CyTOF in primary human cells from adult blood samples (*n* = 3) following 48‐h stimulation with 100 U/mL IFN. (B) Expression of IFITM3 in myeloid and lymphoid cells in adult blood samples following 48‐h stimulation with 100 U/mL IFN. Data analyzed by one‐way ANOVA with Tukey's multiple comparisons test, comparing all IFN stimulations to the no IFN control. (C) Fold change in IFITM3 expression following 48‐h stimulation with 100 U/mL IFN compared to no IFN controls. (D) Flow cytometry plots showing expression of IFITM3 in primary human monocytes (CD14^+^) following up to 72‐h stimulation with 500 U/mL IFN. (E) IFITM3 expression measured by flow cytometry compared to expression 72‐h post IFN stimulation. Data are expressed ±SEM with mean center values. IFN stimulation of adult blood donors *n* = 3, one experiment. Monocyte time‐course *n* = 3, three independent experiments.

Myeloid cells can induce greater than twofold induction with type I IFN, with the exception of CD16^+^ monocytes (Fig. 2C). CD16^+^ monocytes have high basal expression, suggesting that they are already expressing maximal levels of IFITM3. In contrast, we see minimal induction of IFITM3 in lymphoid immune cell subsets (Fig. 2C). NK and B cells show a modest increase in expression following type I IFN stimulation. Neither T cell populations show any increase in IFITM3 expression (Fig. 1B), confirming murine studies showing that only CD3/CD28 activation increased IFITM3 expression [8].

Plasmacytoid dendritic cells (pDC) responded with approximately threefold induction following type III IFN stimulation. These are the only immune cell subset to express the IFN‐λ receptor, IFNLR1, showing that type III IFN can also induce IFITM3 strongly if the receptor is present [9].

The basal expression of IFITM3 is inconsequential if new protein can be induced rapidly during an infection. We found that induction of IFITM3 was not rapid and instead it took ∼36 h to reach maximal IFITM3 expression (Supporting Information Fig. S4). Looking into the timescale of IFITM3 induction in primary cells, we found that expression was highest at 48 h post IFN stimulation in CD14^+^ monocytes (Fig. 2D).

These results show that basal expression of IFITM3 can vary across immune cell subsets, with consistently higher expression on myeloid cells compared to lymphoid. In the myeloid compartment, low basal expression can be overcome by strong type I IFN induction. However, in the lymphoid compartment, there is minimal induction by IFN suggesting that IFITM3 does not play a strong role in viral restriction in these cell types. Knowing that it takes at least 24 h for IFITM3 to be induced strongly by IFN stimulation highlights the importance of basal expression in the early stages of a viral infection.

## Conflict of interest

The authors have no commercial or financial conflict of interest.

### Peer review

The peer review history for this article is available at https://publons.com/publon/10.1002/eji.202048706.

## Supporting information

Supporting information

## Data Availability

The data that support the findings of this study are available from the corresponding author upon reasonable request.
