# Supplementary material for: IFITM3‐specific antibody reveals IFN preferences and slow IFN induction of the antiviral factor IFITM3 in humans
Source: Eur J Immunol. 2020 Dec 9;51(3):742–5. doi: 10.1002/eji.202048706 (PMC7983929; doi:10.1002/eji.202048706)
Supplement: Supplementary file 1 — Supporting information [file EJI-51-742-s001.docx]

**Title: IFITM3-specific antibody reveals interferon preferences and slow interferon induction of the anti-viral factor IFITM3 in humans**

**Supplementary Tables & Figures**

***
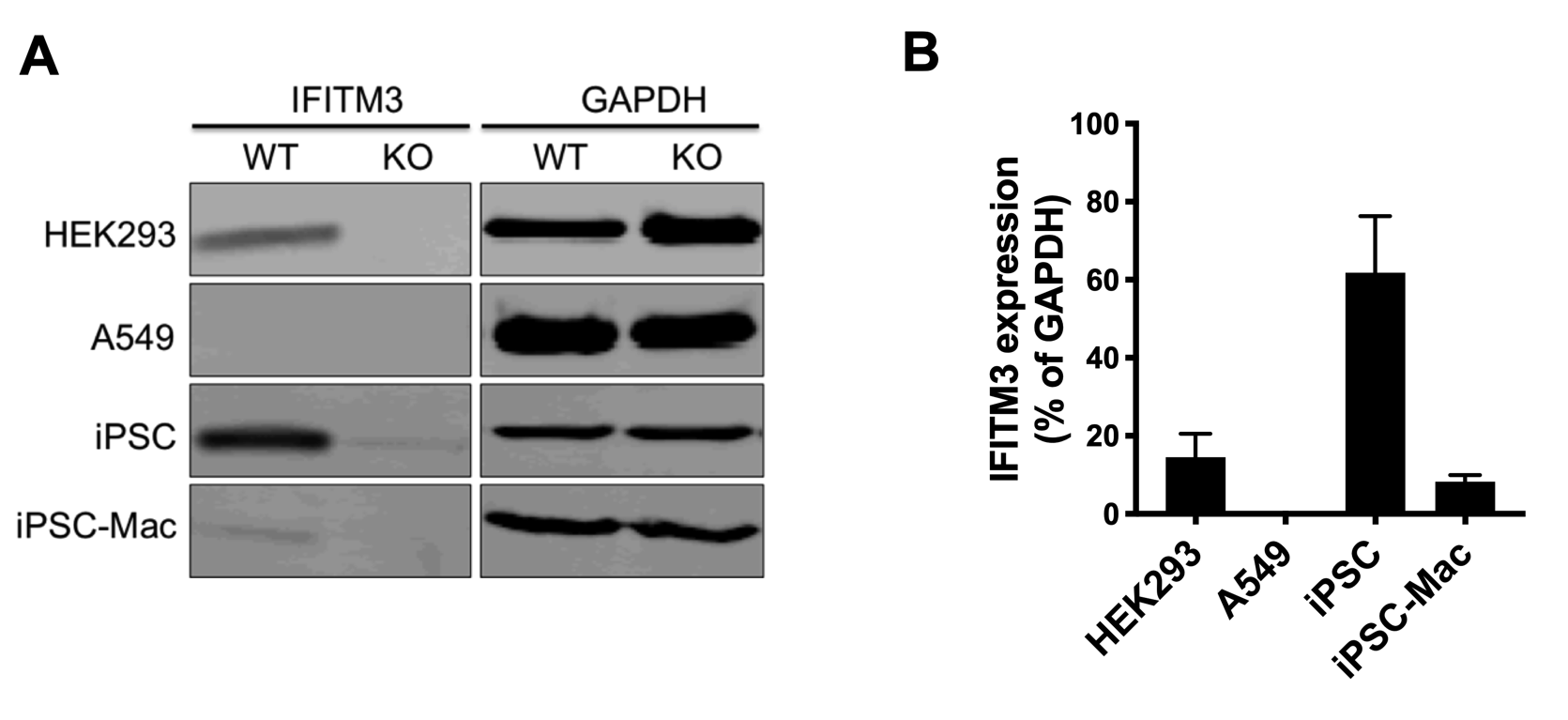
***

***Supplementary Figure 1: IFITM3 expression in WT and IFITM3^-/-^ Cell lines***

(a) IFITM3 protein expression was detected by western blot in cell lysates from WT and IFITM3^-/-^ HEK293, A549, iPSC and iPSC-derived macrophage cell lines. (b) IFITM3 expression in WT cell lines as a percentage of GAPDH expression. Data is expressed as mean values ±SEM, n=3 independent experiments.

***
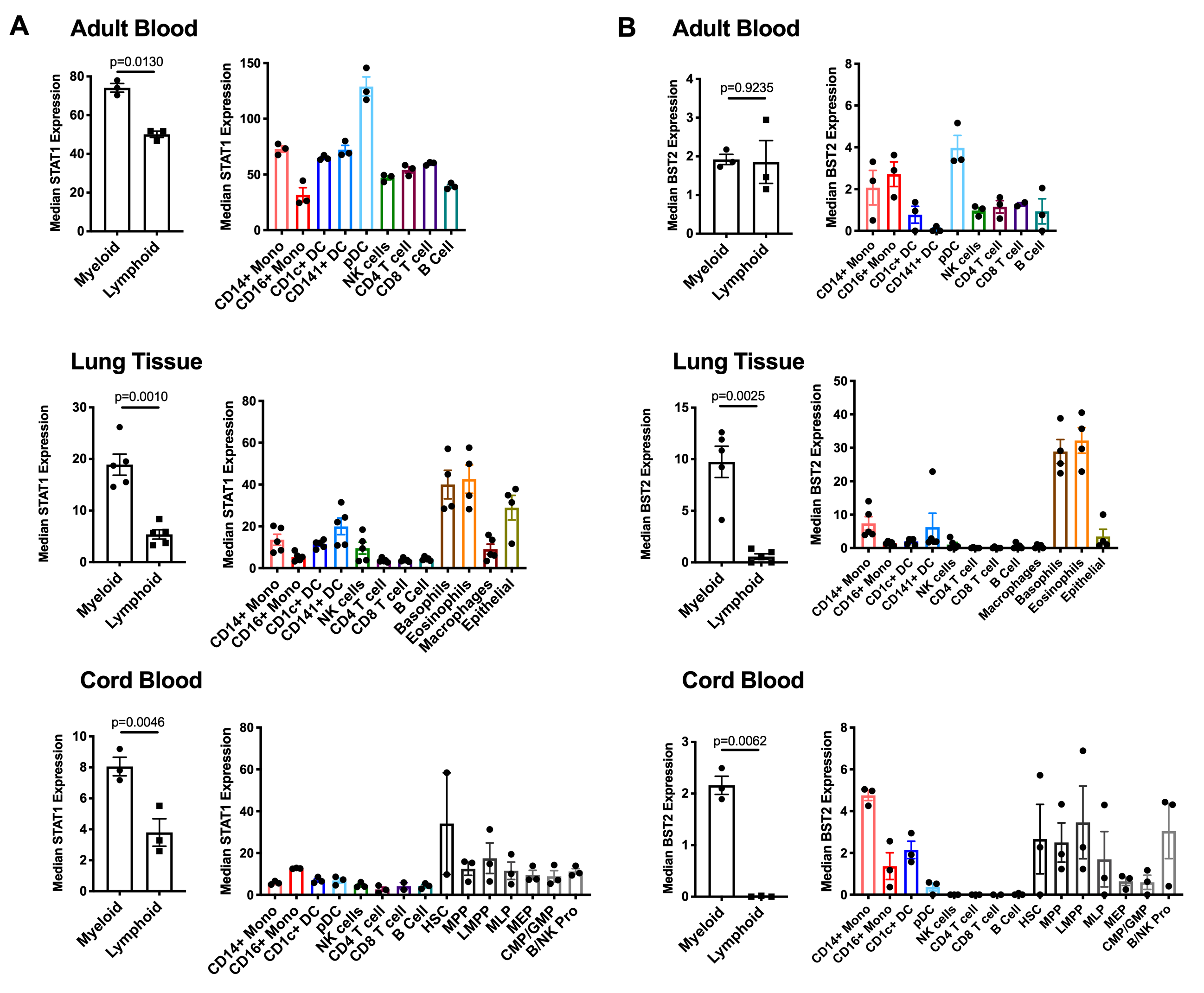
***

***Supplementary Figure 2: Basal expression of STAT1 and BST2 in primary human samples***

(a) Expression of STAT1 was measured by CyTOF in primary human cells from adult blood (n=3), lung para-tumour tissue (n=5) and cord blood (n=3) samples. Data shows myeloid and lymphoid cells grouped as well as individual cell expression. Expression in myeloid and lymphoid cells was compared by paired t-test. (b) Expression of BST2 was measured in primary human cells from adult blood (n=3), lung para-tumour tissue (n=5) and cord blood (n=3) samples. Data shows myeloid and lymphoid cells grouped as well as individual cell expression. Expression in myeloid and lymphoid cells was compared by paired t-test. HSC = hematopoietic stem cells, MPP = multipotent progenitor, LMPP = lymphoid-primed multipotent progenitors, MLP = multi-lymphoid progenitor, MEP = megakaryocyte erythroid progenitor, CMP = common myeloid progenitor, GMP = granulocyte-monocyte progenitors, B/NK Pro = B cell + NK cell progenitor. Data is expressed ±SEM with mean centre values. Adult blood donors n=3, one experiment, lung tissue samples n=5, four independent experiments, cord blood samples n=3, three independent experiments.


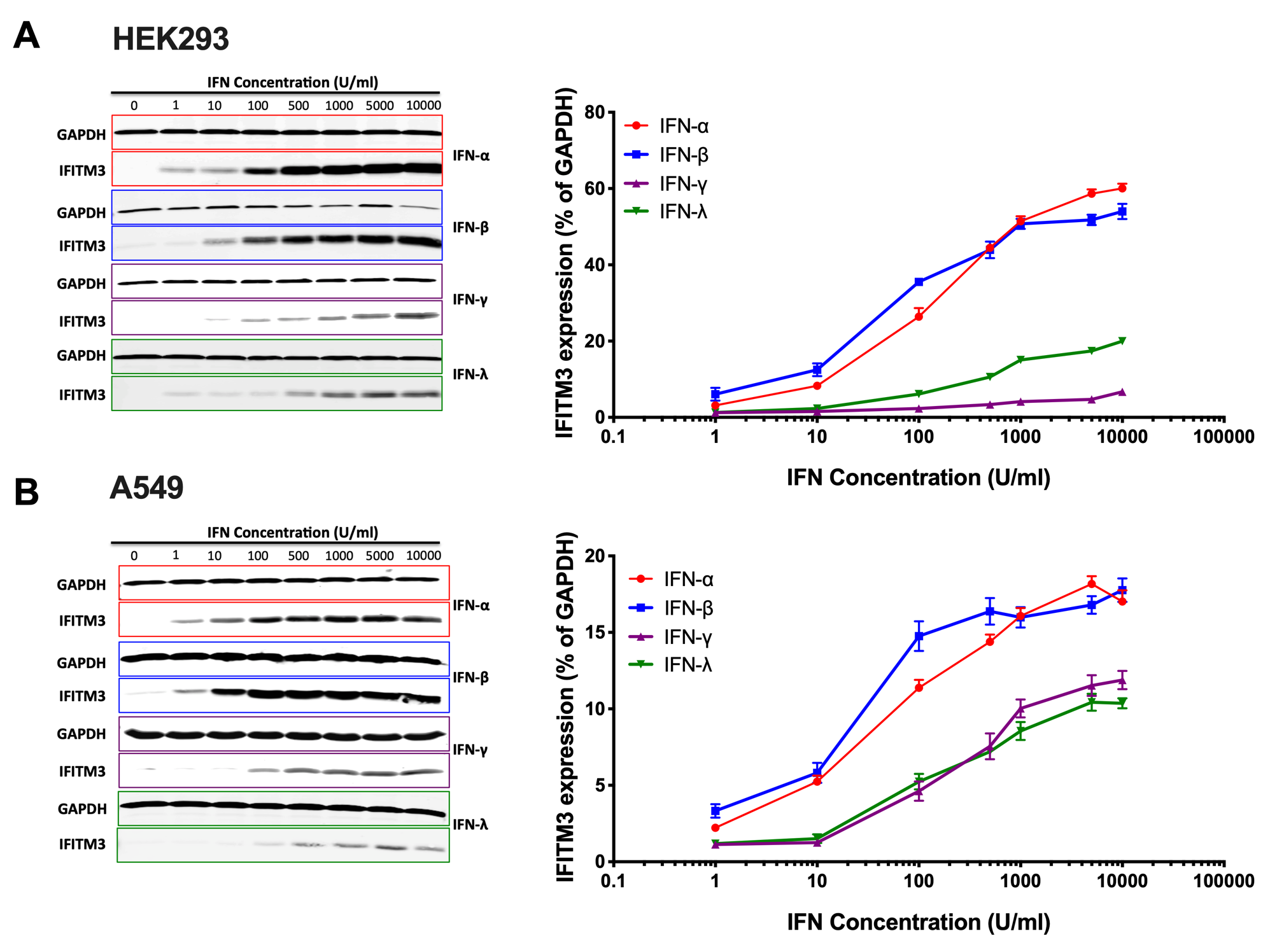


***Supplementary Figure 3: IFITM3 induction by interferon in human cell lines.***

(a) HEK293 cells were stimulated with 0-10,000U/ml interferon for 24 hours prior to measurement of IFITM3 expression by western blot. Data from three independent experiments are presented as a percentage of GAPDH (mean ±SEM). (b) A549 cells were stimulated with 0-10,000U/ml interferon for 24 hours prior to measurement of IFITM3 expression by western blot. Data from three independent experiments are presented as a percentage of GAPDH (mean ±SEM).


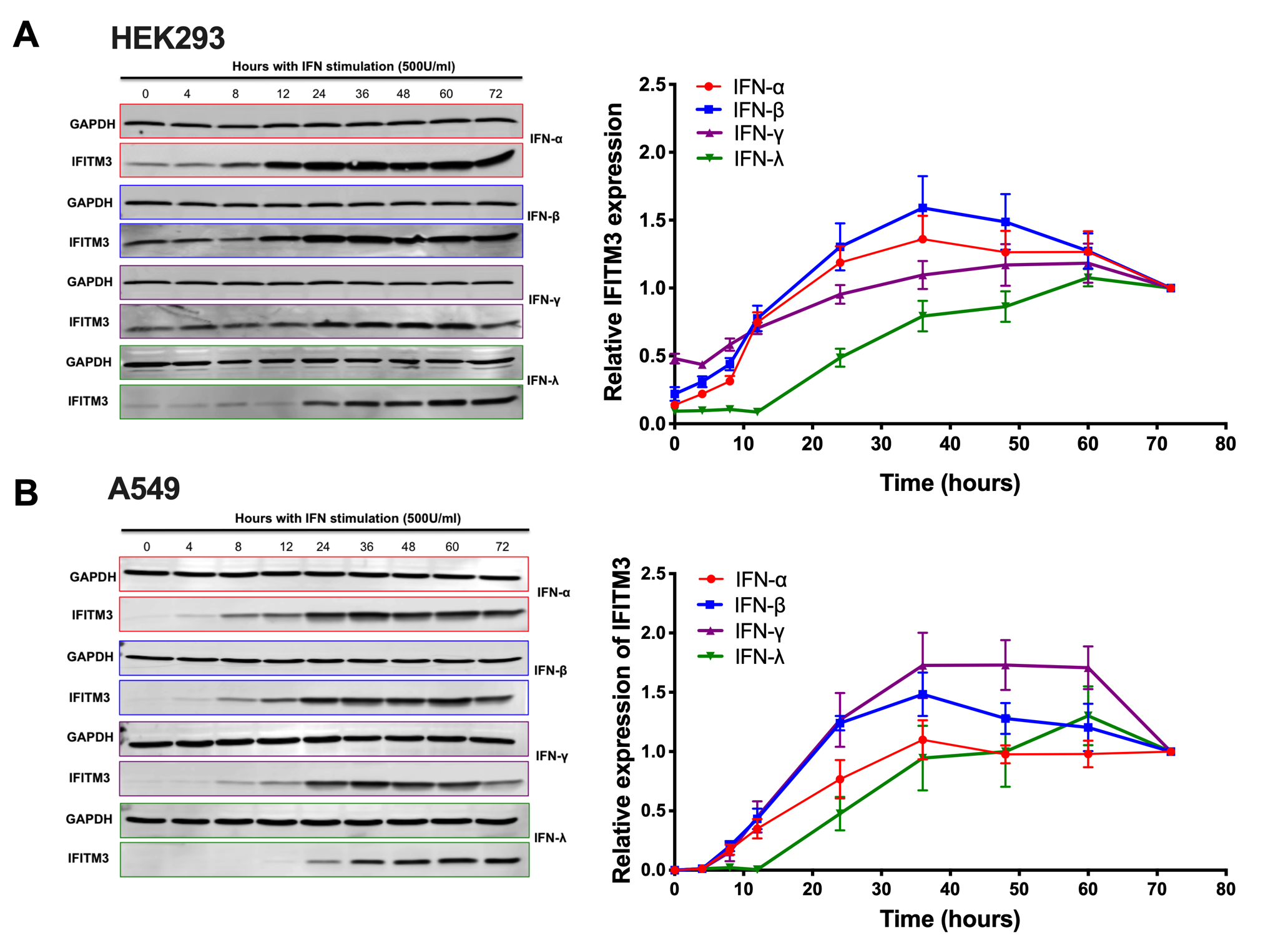


***Supplementary Figure 4: IFITM3 induction over 72 hours following interferon stimulation.***

(a) HEK293 cells were stimulated with 500U/ml interferon for 0-72 hours prior to measurement of IFITM3 expression by western blot. Data from three independent experiments is expressed as a relative level of IFITM3 compared to the level measured at 72 hours (mean ±SEM). (b) A549 cells were stimulated with 500U/ml interferon for 0-72 hours prior to measurement of IFITM3 expression by western blot. Data from three independent experiments is expressed as a relative level of IFITM3 compared to the level measured at 72 hours (mean ±SEM).

**Materials & Methods**

***Study Subjects & Cell culture***

PBMC were isolated from a total of three healthy UK adult volunteers (2 female, 1 male; aged 27-32 years) by Ficoll hypaque separation (Sigma Aldrich). Cord blood from three donors (2 female, 1 male) was obtained through the NHS Blood & Transfusion service and PBMC were isolated as above. Para-tumour lung tissue samples from metastatic cancer or fibrosis patients (total of five donors) were processed using a tumour dissociation kit (MACS Miltenyi Biotec) to isolate immune and epithelial cells. These tissue samples were deemed to show no visible signs of inflammation by a pathologist. The cell lines HEK293, A549 and iPSC were utilised from lab stocks.

PBMC were cultured in RPMI (Lonza) while HEK293 and A549 cells were cultured in DMEM (Sigma). Both were supplemented with 10% foetal calf serum (Sigma), penicillin-streptomycin (Sigma) and 2mM L-glutamine (Sigma). All cells were cultured at 37°C and 5% CO_2_.

The healthy control human iPSC line Kolf2 was acquired through the Human Induced Pluripotent Stem Cells Initiative Consortium (HipSci; [www.hipsci.org](http://www.hipsci.org/)), through which they were also characterized [1]. Consent was obtained for the use of cell lines for the HipSci project from healthy volunteers. A favourable ethical opinion was granted by the National Research Ethics Service (NRES) Research Ethics Committee Yorkshire and The Humber – Leeds West, reference number 15/YH/0391. The Human Induced Pluripotent Stem Cell Initiative was funded by a grant from the Wellcome Trust and Medical Research Council, supported by the Wellcome Trust (WT098051) and the NIHR/Wellcome Trust Clinical Research Facility, and Life Science Technologies Corporation provided Cytotune for reprogramming.

Prior to differentiation, iPSCs were grown feeder-free using the Essential 8 Flex Medium kit (Thermo Fisher Scientific) on Vitronectin (VTN-N, Thermo Fisher Scientific) coated plates as per manufacturer’s instructions to 70-80% confluency. iPSCs were harvested for differentiation using Versene solution (Thermo Fisher Scientific).

***Generation of IFITM3^-/-^ iPSCs***

The Wellcome Trust Sanger Institute core gene-editing pipeline generated IFITM3^-/-^ iPSC lines. The knockout of IFITM3_F01 was generated by a single T base insertion in the first exon using CRISPR/Cas9 in the Kolf2_C1 human iPSC line (a clonal derivative of kolf2 (HipSci)). This was achieved by nucleofection of 10^6^ cells with Cas9**-**crRNA-tracrRNA ribonucleoprotein (RNP) complexes. Synthetic RNA oligonucleotides (target site: 5’- TGGGGCCATACGCACCTTCA CGG, WGE CRISPR ID: 1077000641, 225 pmol crRNA/tracrRNA) were annealed by heating to 95°C for 2 min in duplex buffer (IDT) and cooling slowly, followed by addition of 122 pmol recombinant eSpCas9_1.1 protein (in 10 mM Tris-HCl, pH 7.4, 300 mM NaCl, 0.1 mM EDTA, 1 mM DTT). Complexes were incubated at room temperature for 20 minutes before electroporation. After recovery, cells were plated at single cell density and colonies were picked into 96 well plates. 96 clones were screened for heterozygous and homozygous mutations by high throughput sequencing of amplicons spanning the target site using an Illumina MiSeq instrument. Final cell lines were further validated by Illumina MiSeq. Two homozygous targeted clones were used in downstream differentiation assays.

IFITM3^-/-^ HEK293 and A549 were generated as previously described [2].

***Differentiation of iPSCs to macrophages***

To differentiate iPSCs to iPSC-derived macrophages (iPSC-Mac), the approach of Hale *et al* [3] and van Wilgenburg *et al* [4] was modified. Briefly, upon reaching confluency, human iPSCs were collected and transferred into Essential 8 Flex medium supplemented with 50 ng/mL BMP-4 (Bio-Techne), 20 ng/mL SCF (Bio-Techne) and 50 ng/mL VEGF (Peprotech EC Ltd.) in ultra-low attachment plates (Corning) for 4 days to generate Embryoid Bodies (EBs). On day 5, EBs were used for generation of myeloid precursor cells by plating into 6-well tissue culture treated plates (Corning) coated for two hours at room temperature with 0.1% gelatin, in X-VIVO-15 media supplemented with 25 ng/mL IL-3 (Bio-Techne) and 50 ng/mL M-CSF (Bio-Techne). After several weeks, floating myeloid precursors were harvested and terminally differentiated into matured macrophages in the presence of higher concentrations of M-CSF (100 ng/mL) for 7 days. For protein harvests macrophages were detached using Lidocaine solution (4 mg/mL lidocaine-HCl with 10 mM EDTA in PBS).

**Interferon Stimulation**

All IFNs used were sourced from *PBL Assay Science*. IFN-alpha 2 (Alpha 2b) (Cat.No. 11105-1), IFN-beta 1a (Cat.No. 11415-1), IFN-gamma (Cat.No. 11500-2) and IFN-lambda 3 (IL-28B) (Cat.No.11730-1).

***Western Blot analysis of IFITM3 protein expression***

Western blot for IFITM3 protein expression was performed as described previously [2]. Images from Western blot experiments were analysed by Fiji software for band density and expressed in GraphPad Prism as either a percentage of the GAPDH expression or as a relative amount of IFITM3 compared to a selected time point (72 hours). A one-way ANOVA was performed along with Tukey’s or Sidak’s multiple comparisons tests to measure statistical differences between cell lines.

***Mass Cytometry staining for ISG expression***

Purified antibodies against IFITM3 (in-house clone), STAT1 (clone 246123), CD90 (clone 5E10) and CD38 (clone HIT2) were conjugated in-house using the Maxpar X8 Multi-Metal Labeling Kit (Fluidigm) according to the manufacturer’s instructions. Other antibodies were purchased from the listed sources in **Supplementary table 1**.

To test for reproducibility between CyTOF runs, the same donor was included in each run. 100 mL of heparinized blood was drawn from a healthy control donor, PBMCs were isolated and aliquots were frozen (90% fetal bovine serum+10% dimethyl sulfoxide) and stored in liquid nitrogen until use. With every CyTOF run 1 vial was thawed for staining and acquisition. Analysis of PBMC data across all CyTOF runs showed minimal differences in IFITM3 expression across experiments showing consistent staining and acquisition across experiments (**Supplementary figure 5**).

Donor cells were re-suspended at 1×10^7^ cells/mL and stained with 5 mmol/L Cisplatin (Fluidigm; live/dead) and surface antibody cocktail. Cells were permeabilised with Maxpar nuclear antigen staining buffer and stained with intracellular markers and the metal-conjugated secondary to BST2-PE. An un-permeabilised control without secondary antibody was treated with cell staining buffer and stained with intracellular antibodies. Cells were stained with 125 nM Ir-Intercalator (Fluidigm) according to Fluidigm protocols and fixed with 1.6% formaldehyde. Cells were counted on a BD Accuri C6. Before acquisition on CyTOF Helios cytometer (Fluidigm), cells were re-suspended at 2×10^6^ cells/mL in 0.1×EQ Four Element Calibration Beads (Fluidigm). Data files were processed and normalised using the CyTOF software v6.7 (Fluidigm).

**Mass cytometry Analysis**

CyTOF files (.fcs format) were imported into FlowJo 10.5.2 (Treestar Inc). Live single cells were identified using the gating strategy shown in **Supplementary Figure 5**, along with further gating strategies for the lung and cord blood samples. Myeloid and lymphoid values were calculated from the average of the myeloid (CD14+ monocytes, CD16+ monocytes, CD1c+ DC, pDC and CD141+ DC) or lymphoid (NK cells, B Cells, CD4+ T cells and CD8+ T cells) populations.

For each group (CD34^-^ cord blood PBMC, CD45^+^ lung cells or live adult PBMC) files were downsampled to maximum 250,000 cells per donor or condition, concatenated and exported into one data file. An UMAP analysis was run on each concatenated file using the phenotypic markers. Visualisation of UMAP parameters allowed identification of distinct immune cell subsets (**Supplementary figure 6a-c**). Individual samples were identified by gating on event length v sample ID (**Supplementary figure 6d**). For each individual sample, the median value was determined for IFITM3, BST2 and STAT1. This raw data was plotted on column or grouped graphs in GraphPad Prism. Statistical analysis was completed using paired t-tests.

**Measurement of IFITM3 expression following interferon stimulation**

HEK293 and A549 cell lines were stimulated with 0-10,000U/ml interferon for 24 hours prior to harvest of cells, generation of cell lysates and western blotting for IFITM3 and GAPDH expression, as described above. These cell lines were also cultured for 0-72 hours with 500U/ml interferon prior to harvest, washing in PBS and storage of cell pellets at -20’C until cell lysates for all timepoints could be processed together.

CD14^+^ monocytes were isolated from leukocyte blood cones (NHS Blood & Transfusion service). In brief, PBMC were isolated by Ficoll hypaque separation (Sigma Aldrich) prior to the addition of CD14 magnetic beads (MACS Miltenyi). Positive magnetic separation yielded CD14^+^ populations ranging from x-y% across three independent experiments (**Supplementary figure 7**). These CD14^+^ monocytes were then cultured for 0-72 hours with 500U/ml interferon. At each timepoint (0, 24, 48, 72 hours) cells were harvested, stained with Zombie violet live dead stain (Biolegend), permeabilised (BD Fix & Perm kit) and stained with IFITM3-AF647 (conjugated in-house using Thermofisher AF647 Labelling Kit). Samples were fixed with BD Cell fix and run on the Thermofisher Attune Nxt Flow Cytometer. CD14+ monocytes were identified as Live, single, CD14+ cells, and IFITM3 expression was determined by looking at the Mean fluorescence intensity of IFITM3-AF647 expression (**Supplementary figure 8**).

References:

1. A. Leha, et al., Methods. 2016. 96: 85-96

2. S. Makvandi-Nejad, et al., J Infect Dis. 2017.

3. C. Hale, et al., PLOS ONE. 2015. 10: 5 e0124307

4. B. van Wilgenburg, et al., PLoS One. 2013. 8: 8 e71098

**
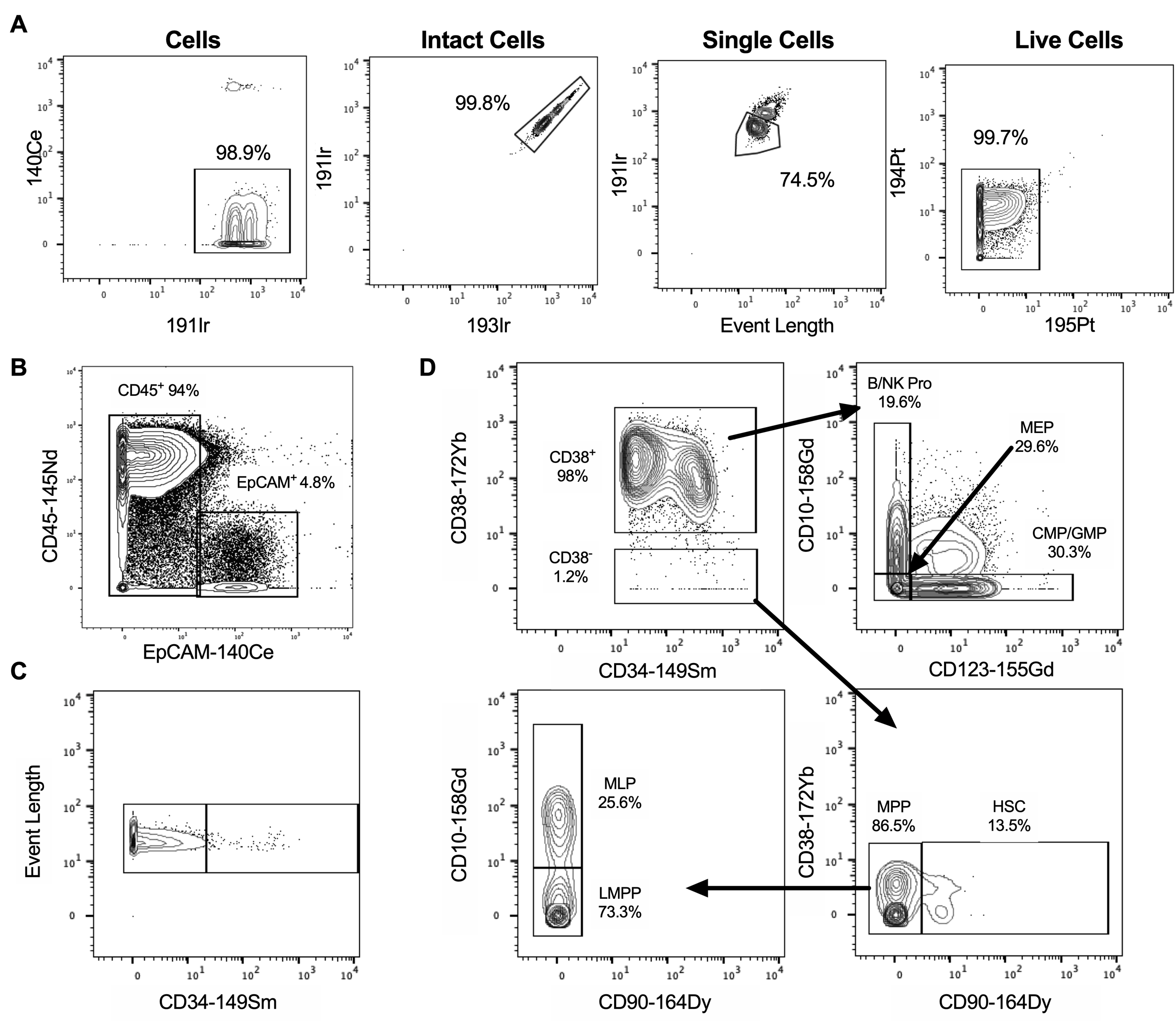
**

***Supplementary Figure 5:* *Mass cytometry gating strategy.***

For analysis of mass cytometry data, we first identified live, singlet, intact cells (a). Adult blood PBMC were then run through UMAP analysis as shown in supplementary figure 6a. Lung tissue samples were further gated to exclude the EpCAM^+^ epithelial cells prior to UMAP analysis (b). Cord blood samples were gated to distinguish the CD34^+^ populations (c) before further sub-gating of this CD34^+^ population as displayed (d). CD34^-^ cells were run through UMAP analysis. HSC = hematopoietic stem cells, MPP = multipotent progenitor, LMPP = lymphoid-primed multipotent progenitors, MLP = multi-lymphoid progenitor, MEP = megakaryocyte erythroid progenitor, CMP = common myeloid progenitor, GMP = granulocyte-monocyte progenitors, B/NK Pro = B cell + NK cell progenitor.

***
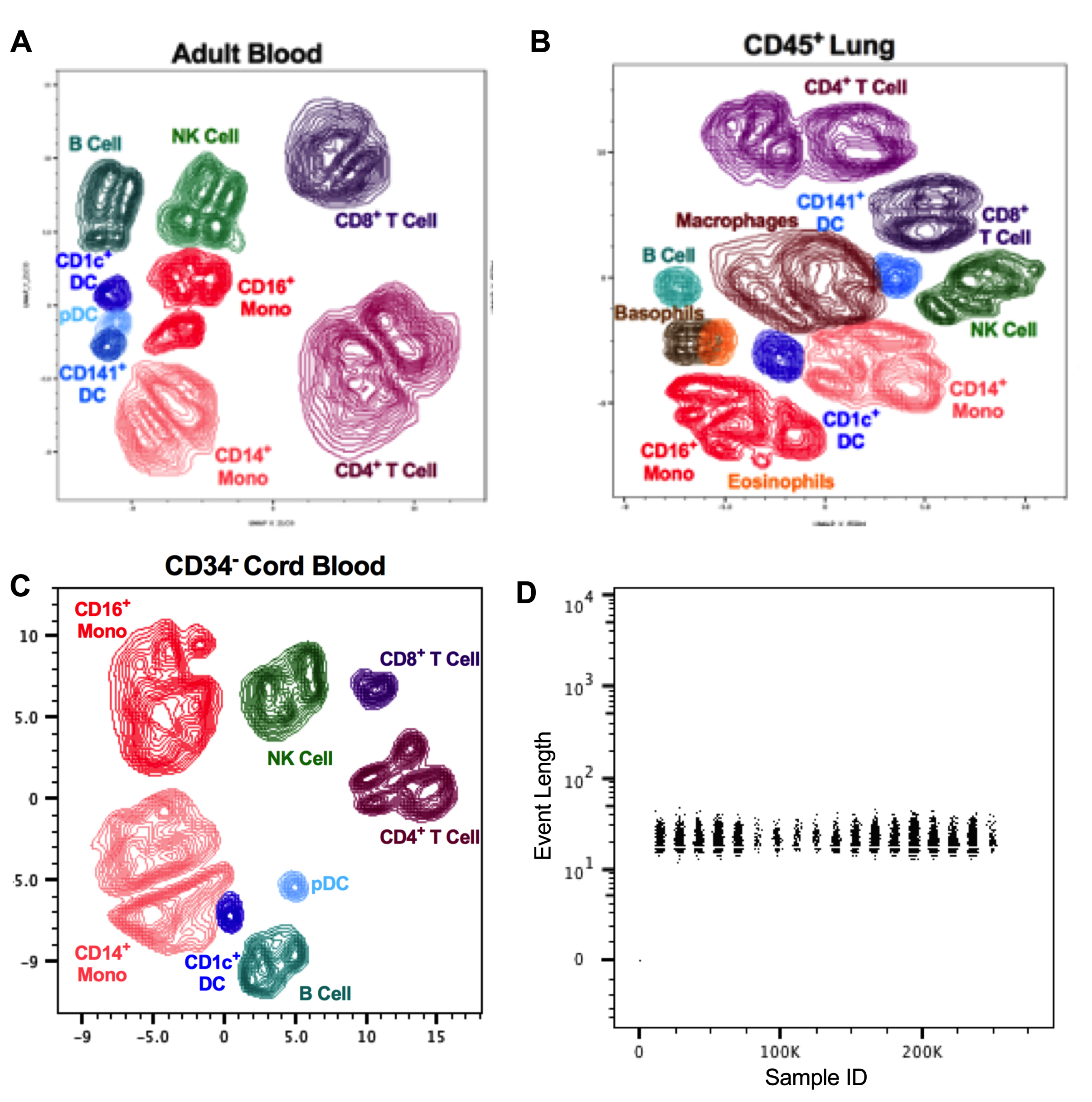
***

***Supplementary Figure 6: Mass cytometry UMAP gating.***

Live, singlet, intact cells for each sample were downsampled using the FlowJo plug-in to 200,000 cells. The downsampled files for each tissue type were concatenated into one file and run through FlowJo UMAP analysis. (a) UMAP output for human adult blood PBMC. (b) UMAP output for CD45^+^ EpCAM^-^ lung tissue cells. (c) UMAP output for CD34^-^ cord blood cells. (d) Following gating on the UMAP output, individual samples can be identified by gating on Event length v Sample ID to reveal individual sample populations.


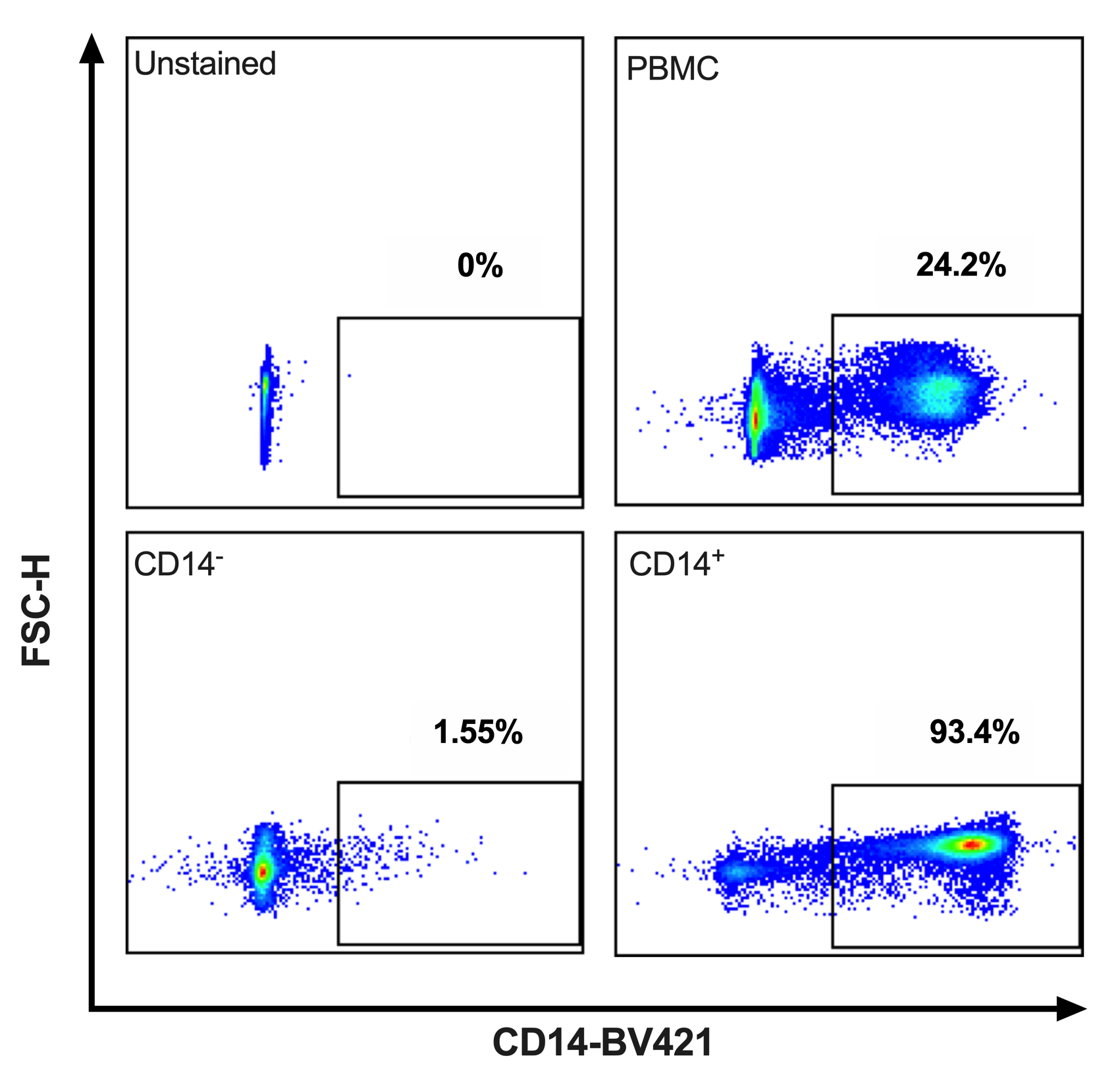


***Supplementary Figure 7: CD14^+^ magnetic bead selection of PBMC.***

CD14^+^ monocytes were isolated from fresh human PBMC samples by positive-selection magnetic bead sorting. The percentage of CD14^+^ live cells is shown for an unstained sample, PBMC prior to sorting, CD14^-^ flow through and the resultant CD14^+^ population. Data representative of one experiment.


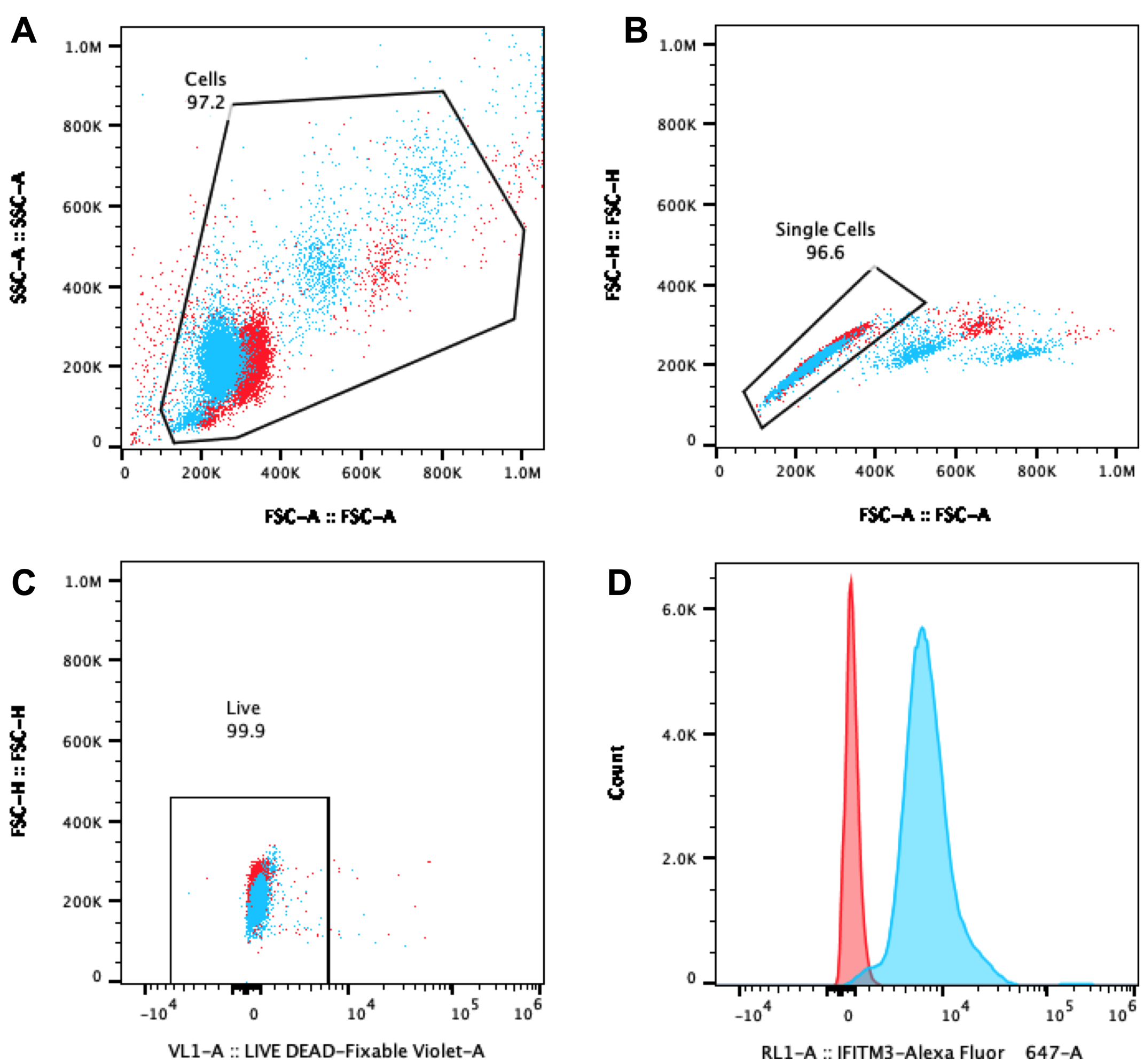


***Supplementary Figure 8: CD14^+^ monocyte gating strategy.***

Cells were identified through SSC-A vs FSC-A gating (A). Single cells were gated on FSC-H vs FSC-A (B). Live cells were identified as LIVE DEAD Fixable Violet negative (C). Mean fluorescent IFITM3-AF647 expression was used to show IFITM3 expression across different conditions. Graphs are representative of monocyte timecourse gating strategy. Red cells are unpermeabilised control and blue cells are permeabilised sample.

***Supplementary table 1: Mass cytometry antibody panel***
